# The Helix Ring Peptide U_11_ from the Venom of the Ant, *Tetramorium bicarinatum*, Acts as a Putative Pore-Forming Toxin

**DOI:** 10.3390/membranes14050114

**Published:** 2024-05-14

**Authors:** Steve Peigneur, Diogo Tibery, Jan Tytgat

**Affiliations:** 1 Toxicology and Pharmacology, University of Leuven (KU Leuven), P.O. Box 922, Herestraat 49, 3000 Leuven, Belgium; steve.peigneur@kuleuven.be (S.P.); dtibery@gmail.com (D.T.); 2 Laboratory of Neuropharmacology, Department of Physiological Sciences, University of Brasília, Distrito Federal, Brasília 70910-900, Brazil

**Keywords:** ant venom, *Tetramorium bicarinatum*, potassium channel, sodium channel, pore-forming toxin

## Abstract

An insect neuroactive helix ring peptide called U_11_-MYRTX-Tb1a (abbreviated as U_11_) from the venom of the ant, *Tetramorium bicarinatum*. U_11_ is a 34-amino-acid peptide that is claimed to be one of the most paralytic peptides ever reported from ant venoms acting against blowflies and honeybees. The peptide features a compact triangular ring helix structure stabilized by a single disulfide bond, which is a unique three-dimensional scaffold among animal venoms. Pharmacological assays using *Drosophila* S2 cells have demonstrated that U_11_ is not cytotoxic but instead suggest that it may modulate potassium channels via the presence of a functional dyad. In our work described here, we have tested this hypothesis by investigating the action of synthetically made U_11_ on a wide array of voltage-gated K and Na channels since it is well known that these channels play a crucial role in the phenomenon of paralysis. Using the *Xenopus laevis* oocyte heterologous expression system and voltage clamp, our results have not shown any modulatory effect of 1 μM U_11_ on the activity of Kv1.1, Kv1.3, Kv1.4, Kv1.5, *Shaker* IR, Kv4.2, Kv7.1, Kv10.1, Kv11.1 and KQT1, nor on DmNav and BgNav. Instead, 10 μM U_11_ caused a quick and irreversible cytolytic effect, identical to the cytotoxic effect caused by *Apis mellifera* venom, which indicates that U_11_ can act as a pore-forming peptide. Interestingly, the paralytic dose (PD_50_) on blowflies and honeybees corresponds with the concentration at which U_11_ displays clear pore-forming activity. In conclusion, our results indicate that the insecticidal and paralytic effects caused by U_11_ may be explained by the putative pore formation of the peptide.

## 1. Introduction

Several thousand species of stinging ants are known, being insects belonging to the family Formicidae and the order Hymenoptera [1]. They are equipped with a venom that contains a true cocktail of bioactive substances [2]. Much like other venomous animals, it is generally assumed that toxins in ant venom are employed for different purposes, such as predation and defense, being designed against bigger animals as well as much smaller microbial pathogens [3]. The observed biological activities associated with ant toxins are impressively diverse and include paralytic, cytolytic, hemolytic, allergenic, pro-inflammatory, insecticidal, antimicrobial, and pain-producing pharmacologic activities [4]. Non-toxic functions have also been reported and include a role in chemical communication involving sex pheromones and deterrents. Antimicrobial peptides (AMPs) are very well known, such as bicarinalin from the ant *Tetramorium bicarinatum* [5] and pilosulin from the ant *Myrmecia pilosula* [6]. They are often characterized by a broad-spectrum activity against Gram-positive and Gram-negative bacteria and against fungal pathogens. Ponericins from the neo-tropical ant *Neoponera commutata* are another example, which are active as anthelmintic agents [6]. Ant venoms are particular and distinct from other animal venoms since they are not only rich in linear and disulfide-rich peptides but also in volatile and non-volatile small molecules such as alkaloids and hydrocarbons. This is highlighted by stingless ants from the subfamily Formicinae, which spray their venoms through a special opening called the acidopore, with the well-known formic acid molecule as a key example, and its name is derived from this (sub)family [for a review, see [4]].

Ant venom peptides have previously been classified based on their structure into three main groups: linear, dimeric or inhibitor cystine knot (ICK)-like peptides [for a review, see [2]]. An alternative approach for classification follows their biological activity, with two main characteristics: cytolytic or neurotoxic. The wide prevalence of small linear peptides devoid of disulfide bonds, smaller than 35 residues, and with cytolytic activity has clearly been demonstrated in ant venoms. They often act as amphipathic, helical structures forming pores through biological membranes, disturbing the cellular integrity and facilitating the passage of other disulfide-rich neurotoxins to their molecular targets [7]. In this manner, cytolytic peptides act synergistically with neurotoxins, a phenomenon also observed in spiders. As compared to other venomous animals, studies investigating neurotoxic ant venom peptides are relatively scarce. The best-known examples are (*i*) poneratoxin, a small 25-residue peptide derived from the bullet ant *Paraponera clavate*; it has been shown to modulate voltage-gated sodium (Nav) channels of both vertebrates and invertebrates, blocking synaptic transmission in the insect CNS [8,9], and (*ii*) ectatomin Et-1, from the ant *Ectatomma tuberculatum* [10], which is a voltage-gated calcium (Cav) channel blocker and also a pore-forming peptide cytotoxic to vertebrate and invertebrate cells [11,12]. This dual pharmacological feature is an interesting observation and will be discussed further, given its relevance for this work. Different from pilosulins and ectatomins, myrmexins have been described as heterodimeric peptides isolated from the venom of *Pseudomyrmex triplarinus* with an anti-inflammatory function [13]. In a transcriptome analysis of the venom glands of the giant ant *Dinoponera quadriceps*, another structural class of ant venom peptides was discovered: ICK-like peptides [14]. They contain three disulfide bonds forming a pseudo knot and are very stable. They are also often present in the venoms of cone snails and spiders and typically have neurotoxic properties. They have proven to be great leads in drug discovery [15]. Unfortunately, in the case of several disulfide-rich ant venom peptides, their role and biological target remain unknown at present. Apart from the presence of peptides in ant venom, accumulating data become available indicating the presence and importance of a number of proteins that, among other effects, cause neurotoxicity [for a review, see [4]]. For example, in the venom of the fire ant *Solenopsis invicta*, a protein homologous to U5-ctenitoxin-Pk1a-like protein, found in the venom of the spider *Phoneutria nigriventer*, has been reported [16] and is involved in causing spastic paralysis in mice [17]. Furthermore, phospholipases have been described as important larger proteins present in several hymenopteran venoms and are considered potent neurotoxic, cytotoxic and allergenic proteins.

In more recent studies published since 2020, Touchard et al., using an integrated transcriptomic and proteomic approach, characterized the venom peptidome of the European red ant, *Manica rubida*. They identified 13 myrmicitoxins that share sequence similarities with previously identified ant venom peptides, one of them being identified as an EGF-like toxin. Insecticidal assays of reversed-phase HPLC venom fractions on the blowfly *Lucilia caesar* enabled the authors to identify six myrmicitoxins with insecticidal activity. It was concluded that *M. rubida* employs a paralytic venom rich in linear insecticidal peptides, which likely act by disrupting cell membranes [18]. In 2021, Robinson et al. characterized an O-linked glycopeptide, Mg7a, as a major component of the venom of the ant *Myrmecia gulosa*. The authors showed that Mg7a is paralytic and lethal to insects and triggers pain behavior and inflammation in mammals, which it achieves through a membrane-targeting mode of action. Interestingly, the deglycosylation of Mg7a renders it insoluble in aqueous solution, suggesting a key solubilizing role of the O-glycans [19]. Last year, Barassé et al. studied peptide U_11_ from *Tetramorium bicarinatum*, a 34-amino-acid peptide, with in-depth insecticidal, structural and pharmacological experiments [20]. They concluded that U_11_ is one of the most paralytic peptides ever reported from ant venoms against blowflies and is also capable of paralyzing honeybees. An NMR spectroscopy of U_11_ uncovered a unique scaffold featuring a compact triangular ring helix structure stabilized by a single disulfide bond. Pharmacological assays using *Drosophila* S2 cells demonstrated that U_11_ is not cytotoxic but suggest that it may modulate potassium conductance, which structural data seem to corroborate [20].

## 2. Materials and Methods

### 2.1. U_11_ Peptide Synthesis and Analytical Validation

**Figure 1:**
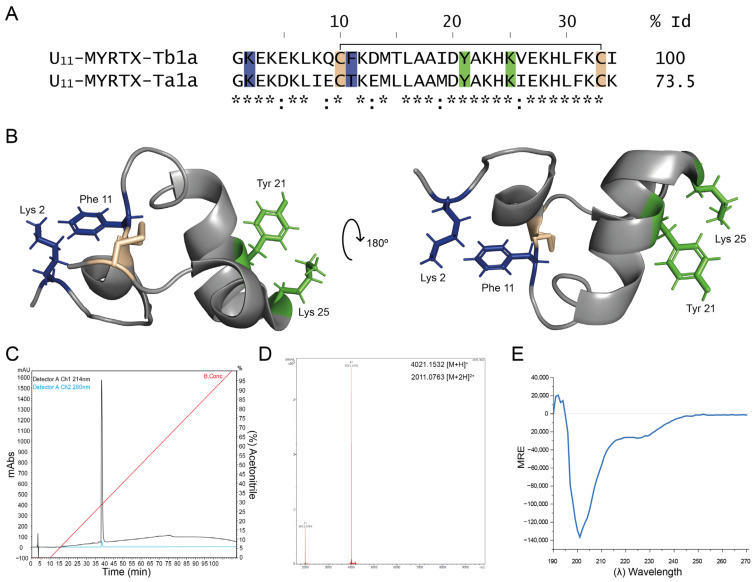
(**A**) Sequence alignment of two described U_11_ peptides. The * indicates identical residues. (**B**) Three-dimensional ribbon representation of U11-MYRTX-Tb1a, (PDB: 8PWT). Side chains of previously proposed two functional dyads are colored in blue (Lys2-Phe11) and green (Tyr21-Lys25). The Cys residues are colored in wheat. (**C**) Chromatographic profile of U_11_ synthetic peptide using RP-HPLC with a C18 column. (**D**) Monoisotopic mass of 4021.1532, expressed as [M+H]^+^, and 2011.0763, expressed as [M+2H]^2+^. (**E**) Circular dichroism (CD) spectrum.

10 mg of >95% pure U_11_ was synthesized (Biomatik, ON, Canada) with free amine and acid termini, respectively. The amino acid sequence is shown in Figure 1A. Peptide purity was assessed in-house prior to testing its bioactivity by analytical reversed-phase high-performance liquid chromatography (RP-HPLC) using a Shim-pack Arata C18 column (dimensions of 4.6 mm × 250 mm and particle size of 5 µm). The column was equilibrated with an isocratic elution of 100% solvent A and 0.1% trifluoroacetic acid (TFA) in water for 10 min. The equilibration was followed by a linear gradient from 0% to 80% of solution B and 0.1% TFA in acetonitrile (ACN) over 80 min, ending with an isocratic elution of 80% B for 10 min. The absorbance was monitored at wavelengths of 214 nm and 280 nm. Figure 1C shows a clear, single and symmetrical peak. No impurities or degradation products were observed. U_11_ was dissolved in ND-96 (see further) prior to testing and added to the bath solution of the recording chamber. Using a high-resolution timsTOF Flex instrument in the MALDI positive mode (Bruker, Billerica, MA, USA), Figure 1D illustrates a monoisotopic mass of 4021.1532, expressed as [M+H]+, indicating that the peptide we have used in our functional tests corresponds with oxidized U11 (thus with the disulfide bridge formed); the analytical result of the [M+2H]2+ monoisotopic mass corresponds with 2011.0830. Figure 1E shows circular dichroism (CD) results (J-1500-150ST CD Spectrometer, Jasco, Kingsgrove, NSW, Australia) of U11, proving correct folding with the predicted alpha-helical structures present.

### 2.2. Expression of Ion Channels in Xenopus Laevis Oocytes

The following genes encoding ion channel subunits were expressed in *Xenopus laevis* oocytes: for voltage-gated potassium channels, Kv (rKv1.1, hKv1.3, rKv1.4, rKv1.5, rKv4.2, hKv7.1, hKv10.1, hKv11.1, *Shaker*-IR from *Drosophila melanogaster* and KQT-1 from *Caenorhabditis elegans)* and for voltage-gated sodium channels, Nav (the arthropod channels BgNav1 from *Blattella germanica DmNav1* and TipE from *D. melanogaster*).

Linearized plasmids bearing the ion channel genes were transcribed using the mMESSAGE mMACHINE SP6 or T7 transcription kits (Ambion, Austin, TX, USA) to prepare the respective cRNA. The harvesting of stage V–VI oocytes from anesthetized female *Xenopus laevis* frogs was described previously [21,22]. Oocytes were injected with 50 nL of cRNA at a concentration of 1 ng nL^−1^ using a micro-injector (Drummond Scientific, Broomall, PA, USA). The oocytes were incubated at 16 °C in ND96 solution containing (in mM): NaCl, 96; KCl, 2; CaCl_2_, 1.8; MgCl_2_, 2; and HEPES, 5 (pH 7.4), supplemented with 50 mg L^−1^ gentamicin sulfate.

### 2.3. Two-Electrode Voltage Clamp

Recordings were performed at room temperature (18 °C–22 °C) using a Geneclamp 500 amplifier (Molecular Devices, San Jose, CA, USA) controlled by a pClamp data acquisition system (Axon Instruments, San Jose, CA USA). Whole-cell currents from oocytes were recorded 1–4 days after cRNA injection. The bath solution composition was ND96. Voltage and current electrodes were filled with 3 M KCl. Resistances of both electrodes were kept at 0.7–1.5 MΩ. Elicited currents were sampled at 1 kHz and filtered at 0.5 kHz (for potassium currents) or sampled at 20 kHz and filtered at 2 kHz (for sodium currents) using a four-pole low-pass Bessel filter. Leak subtraction was performed using a−P/4 protocol.

In order to check whether the venoms were cytotoxic to oocytes or not, we used non-injected oocytes without expressing any particular type of ion channel. For the electrophysiological measurements, we used a 2 s ramp protocol from −120 to +80 mV, with cells clamped at −120 mV holding potential. Kv1.x currents were evoked by 500 ms step depolarizations to 0 mV, followed by a 500 ms pulse to −50 mV from a holding potential of −90 mV. Kv4.2, Kv7.x and KQT1 currents were elicited by 700 ms pulses to +20 mV from a holding potential of −90 mV. Current traces of the Kv11.1 channel were elicited by applying a +40 mV prepulse for 2 s, followed by a step of −120 mV for 2 s. Current traces of Kv10.1 were elicited by a 2 s depolarization to 0 mV from a holding potential of −90 mV. Sodium current traces were evoked by a 100 ms depolarization to 0 mV. The current–voltage relationships were determined by 100 (Nav) or 500 (Kv) ms step depolarizations between −90 and +65 mV using 5 (Nav) or 10 (Kv) mV increments. Toxin-induced effects on the Nav channel steady-state inactivation were investigated by using a standard 2-step protocol. In this protocol, 100 ms conditioning 5 mV step prepulses ranging from −90 to +70 mV were followed by a 50 ms test pulse to 0 mV. Data were normalized to the maximal Na^+^ current amplitude, plotted against prepulse potential and fitted using a Boltzmann function. All data were obtained in at least five independent experiments (*n* ≥ 5). Animal experiments using *Xenopus laevis* were approved by the Animal Ethics Committee of KU Leuven in accordance with EU Council Directive 2010/63/EU.

## 3. Results

Peptide U_11_-MYRTX-Tb1a, abbreviated as U_11_ with ‘U’ referring to ‘unknown function’, from the venom of the ant *Tetramorium bicarinatum*, is a 34-amino-acid peptide containing a single disulfide bond. A closely related peptide from the ant *Tetramorium africanum* was also reported [23], named peptide U_11_-MYRTX-Ta1a, with 73.5% identity to U_11_ and investigated by us here (Figure 1A). NMR spectroscopy carried out by Barassé et al. (2023) revealed a unique scaffold for U_11_ with a compact triangular ring helix structure stabilized by this disulfide bridge (Figure 1B). Interestingly, several pore-blocking peptides of voltage-gated K channels have been described from different venomous animals, where two crucial residues, commonly known as ‘functional dyad’, take part in physically occluding the pore of these channels [24]; this has also been shown by our group—see [25]. The dyad is composed of a basic amino acid, lysine, protruding into the selectivity filter of the K channel and stabilized by a hydrophobic aromatic amino acid, usually a tyrosine or phenylalanine residue. Barassé et al. (2023) [20] have noticed in their work the presence of two potential dyads in U_11_: Lys2-Phe11 and Lys25-Tyr21. Residues of these functional dyads are illustrated in a ribbon presentation in blue and green (Figure 1B). Since residue Phe11 is not conserved between the two *Tetramorium* species, Barassé et al. (2023) [20] suggested that Lys25-Tyr21 appears more likely as the putative pharmacophore. This hypothesis was further strengthened by the observation that it superimposes the dyad in the sea anemone peptide BgK (PDB: 1BGK), with a similar distance of approximately 7.2 Å between the two residues. BgK is a peptide with the ShKT fold that is widely distributed in nature. Many of these peptides block voltage-gated K channels with interesting selectivity and/or impressive affinity [26]. The obvious presence of such putative pharmacophore, as hypothesized by Barassé et al. (2023) [20], was the rationale for us to check whether U_11_ is indeed able to block voltage-gated K channels, and if so, which one(s) and how potently? In the next section, we have therefore undertaken an extensive screening of many types of voltage-gated K channels.

**Figure 2:**
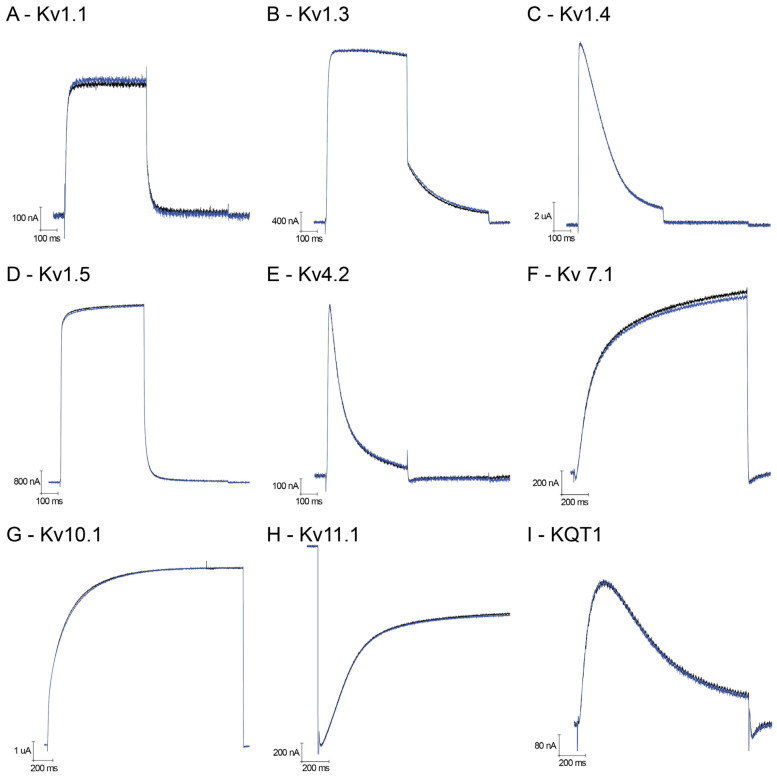
Electrophysiological characterization of U_11_ on voltage-gated potassium channels. (**A**–**I**) Representative traces of evoked current. (**A**) Kv1.1; (**B**) Kv1.3; (**C**) Kv1.4; (**D**) Kv1.5; (**E**) Kv4.2; (**F**) Kv7.1; (**G**) Kv10.1; (**H**) Kv11.1; and (**I**) KQT1.

**Figure 3:**
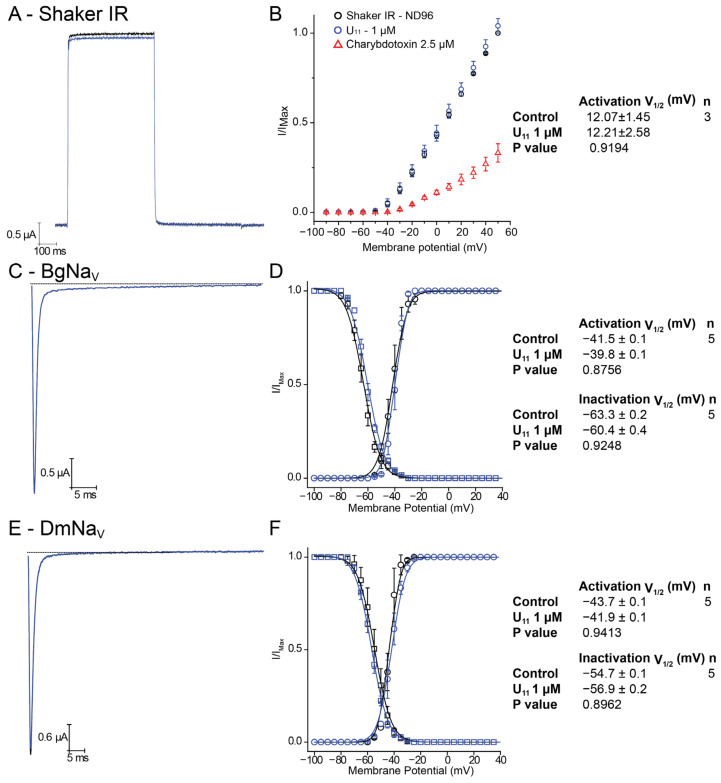
Electrophysiological characterization of U_11_ on voltage-gated invertebrate potassium and sodium channels. (**A**) Outward potassium currents of Shaker IR, in control (black trace) and 1 μM condition (blue trace). (**B**) Shaker IR current–voltage relationship in control (black circles), 1 μM U_11_ (blue circles) and 2.5 μM charybdotoxin (red triangles). (**C**) Inward sodium currents of BgNav. (**D**) Steady-state activation (black circles represent control condition and blue circles represent U_11_ 1 μM condition) and steady-state inactivation (black squares represent control condition and blue squares represent U_11_ 1 μM condition) for BgNaV. (**E**) Inward sodium currents of DmNav. (**F**) Steady-state activation (black circles represent control condition and blue circles represent U_11_ 1 μM condition) and steady-state inactivation (black squares represent control condition and blue squares represent U_11_ 1 μM condition) for DmNaV. The insets on the right summarize for each panel the obtained values and number of tested cells.

Using the heterologous *Xenopus laevis* expression system, we have been able to functionally study voltage-gated potassium channels belonging to different families: from the Shaker family, Kv1.1, Kv1.3, Kv1.4, Kv1.5 and *Shaker* IR (‘Inactivation Removed’); from the *Shal* family, Kv4.2; and together with Kv10.1 (known as *EAG*), Kv11.1 (known as *ERG*) and KQT-1. Figure 2A–I show representative traces of outward potassium currents evoked during step depolarizations, where some of these channels inactivate during the test pulse; for Kv11.1, inward tail potassium currents are shown, representing recovery from inactivation typically seen with this channel. In all cases, the presence of 1 μM U_11_ did not induce any effect (neither a block nor an increase in the K current). As a control, we have compared the lack of effect of U_11_, again at 1 μM on the *Shaker* IR channel with the well-known charybdotoxin at 2.5 μM: as expected, a clear block of *Shaker* IR currents is seen in the presence of charybdotoxin in contrast to U_11_ (Figure 3A).

Since Barassé et al. (2023) stated that U_11_, in their hands, is the most paralytic peptide ever reported from ant venoms against blowflies, is also capable of paralyzing honeybees and it has received little attention yet holds great promise for the discovery of novel insecticidal molecules, we have also checked whether the observed paralysis seen in blowflies and honeybees could be explained via modulation of voltage-gated sodium channels. Historically, it is well known that voltage-gated sodium channels are a key target for several classes of insecticides [for a review, see [27]]. Figure 3 shows representative inward sodium currents evoked by a step depolarization of the insect cockroach sodium channel BgNav1 (panel A) and the fruit fly DmNav1 (panel C). Application of 1 μM U_11_ again did not show any effect. A careful analysis of the steady-state Boltzmann activation and inactivation curves also did not reveal any shift or change (panels B and D).

**Figure 4:**
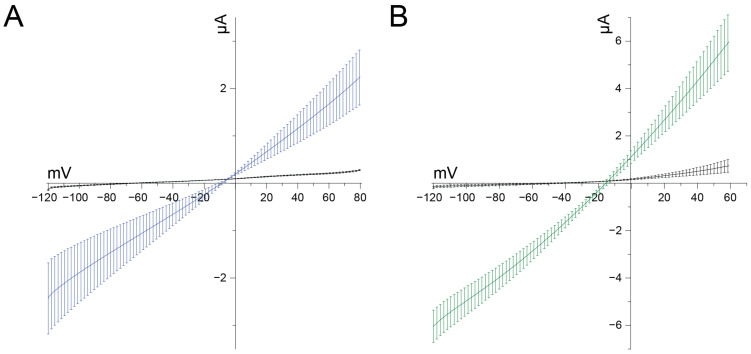
(**A**) U_11_ 10 μM affects reversal potential as a putative pore-forming peptide. Currents recorded from non-injected control oocytes stimulated with voltage ramp protocols of 2 s from −120 to +80 mV. Black trace represents control condition recorded with standard ND96; blue trace represents 10 uM U_11_ condition (n = 8). (**B**) *Apis mellifera* venom in 5 ug/mL condition (green traces) with control (black traces) (n = 5).

In the course of our screenings, we noticed that upon application of concentrations of U_11_ higher than 1 μM, the oocytes quickly became leaky and died soon after. Such a phenomenon is indicative of cytolysis or cytotoxicity and may be explained by pore formation. To investigate this in detail, we compared the effect of 10 μM U_11_ with crude venom of the honey bee *Apis mellifera* since this bee venom is well known for bioactive substances causing cytolysis via a process of pore formation, among which is the peptide melittin [for a review, see [28]]. In non-injected control oocytes, the current evoked by a voltage ramp is minimal in amplitude, and the slope is minimal (small chord conductance) (Figure 4). A reversal potential of −67.5 mV can be noticed, indicating a healthy condition of the oocyte. The application of 10 μM U_11_ quickly and irreversibly caused two clear effects: (1) a shift of the reversal potential in the depolarizing direction to −12.63 mV, and (2) a significant increase in slope (increased chord conductance). Interestingly, a similar effect was obtained with crude venom from *Apis mellifera*, with a shift of the reversal potential to −14.96 mV and a dramatic increase in slope. These observations can be explained by a putative pore-forming mechanism caused by U_11_ and bee venom, and its relevance in the context of U_11_ and its insecticidal action will be discussed in detail below.

## 4. Discussion

Stinging ants of different subfamilies, such as Myrmicinae, possess complex venom mixtures rich in hyaluronidase, phospholipases, peptidyl toxins, histamine and further low-molecular-mass compounds [for a review, see [7]]. All these molecules interact together, resulting in extremely rapid immobilization and/or killing of prey or aggressor. Most proteomic studies on ant venoms have confirmed the prevalence of small linear peptides (devoid of disulfide bonds) with fewer than 35 residues [for a review, see [4]]. These small peptides are often cytolytic with insecticidal, hemolytic and/or antimicrobial properties. Examples include ponericins from the ant *Neoponera goeldii* that exhibit hemolytic activity and antibacterial activity against both Gram-positive and Gram-negative bacteria, as well as insecticidal activity. Additional homologous toxins include the bicarinalins from *Tetramorium bicarinatum* (Myrmicinae), the species of our study. Another group of ant venom peptides are pilosulins from *Myrmecia pilosula* (Myrmeciinae). For instance, pilosulin 1 is a 57-amino-acid-long linear peptide with hemolytic and cytolytic activities. Although several of these peptides are presented as allergens, their exact biological function and mechanism of action often remain unknown. In spider and scorpion venoms, cytolytic peptides are believed to act as membrane-disrupting agents, commonly known as pore-forming peptides, facilitating the passage of other bioactive molecules through cellular barriers. Currently, several mechanisms promoting pore formation have been proposed in the literature, with the most well-documented being the toroidal model, the barrel-stave model, the carpet model and the detergent model [29,30]. Pore formation leads to the quick breakdown of the transmembrane potential by the loss of ion gradients, resulting in cellular death, on the basis of which these peptides are called cytolytic or cytotoxic. The type of pore formed and its dimensions are relevant in this respect. Different strategies exist that can be used to estimate pore dimensions formed by the peptide [29], such as liposome leakage experiments, advanced microscopy, neutron or X-ray scattering and electrophysiological techniques, with the last strategy being used by us and explained further.

Since our results obtained with U_11_ point in the direction of putative pore formation, let us now elaborate first on this aspect below. Parker and Feil (2005) state that pore-forming toxins (PFTs) are one of nature’s most potent biological weapons [31]. A unique feature is the remarkable property that PFTs can exist either in a stable water-soluble state (without much toxicity involved) or as an integral membrane pore (with clear toxicity). In order to convert from the water-soluble to the membrane state, the toxin undergoes a large conformational change driven by the proximity and composition of a barrier, such as a phospholipid cell membrane. Notwithstanding the fact that PFTs differ markedly in their primary, secondary and tertiary structures, they can be classified into one of two families based on the types of pores they form: alpha-PFTs or beta-PFTs. Two points of view need to be discussed: qualitative and quantitative.

From a qualitative point of view, the observation that U_11_ can form putative pores is novel, given its 3-dimensional structure. We have no evidence whether or not U_11_ needs to polymerize in order to form a pore, but the HPLC profile does not indicate the presence of multimers in the presence of the solutions used in the chromatography (Figure 1C). Evidently, on the one hand, this does not exclude possible multimerization in case U_11_ is exposed to a phospholipid membrane, but on the other hand, it seems unlikely that a significant conformational change would take place in the case of U_11_ due to its rigid and cysteine-stabilized scaffold, in contrast to what is observed with cysteine-free linear cytolytic peptides. Interestingly, the presence in U_11_ of two 3_10_-helical segments (Leu7 to Gln9 and Glu27 to Leu30) located at either end of the alpha-helical stretch (Ala17 to His24) mimics a conformational preference also found in pore-forming molecules such as alamethicin [32]. The preference consists of the short bits of a 3_10_-helix tightening up the ends of the alpha-helix by moving the related peptide groups nearer the axis. Barassé et al. (2023) indicated in their work that residues Cys10 to Cys33 delineate an almost planar triangular monocycle closed by the disulfide bridge [20]. Future studies are needed to investigate whether this structural feature is possibly involved in a phenomenon of U_11_’s multimerization, leading to pore formation once a critical concentration is reached. Based on the HPLC-based purity check we carried out of our synthetic U_11_, we have no indication that possible degradation products would be responsible for the observed pore formation.

From a quantitative point of view, we have found an interesting correlation between the reported neurotoxicity by Barassé et al. (2023) on the insect species and cells they have used and the putative pore formation we observe: Barassé et al. report that 60% of blowflies were affected 24 h post ingestion with a dose of 8 mg/mL (i.e., 350 nmol·g^−1^). Taking into account the molecular mass of U_11_ (4018.1), this is 2 μM. Interestingly, we have used in our oocyte assay either 1 μM or 10 μM: with 1 μM, no effects were observed on the ion channels tested, and with 10 μM, a clear cytolytic effect was present. In other words, the 2 μM reported by Barassé et al. falls in between and confirms that we have used the same range of concentrations. Moreover, it is striking that the percentage paralysis observed by Barassé et al. in both *L. caesar* and *A. mellifera* insects, expressed as 50% paralyzing dose (PD50), matches exactly with the concentration range at which U_11_ seems to be able to form pores. The weak effect seen with U_11_ in the experiments by Barassé et al. in the aphid *A. pisum* is difficult to compare, in our view, with the effect seen in the other two insects, since the injection route was different: intrathoracic injection vs. intra-abdominal injection. It is not illogical that the route of administration crucially determines the final effect. In this way, we acknowledge that it is difficult to compare the doses/concentrations/duration of U_11_ used in Barassé’s insect experiments and those used here on *Xenopus* oocytes. The smaller effect on relative fluorescence induced by (a high concentration of) 10 μM U_11_ using *Drosophila* S2 cells, as seen by Barassé et al., could be explained by the fact that U_11_ already provokes a membrane depolarization due to pore formation prior to the application of 50 mM KCl, resulting in a lower relative fluorescence signal.

At this stage, we cannot exclude the possibility that the membrane composition of oocytes of *Xenopus laevis* is so much different from the ones in insects, and as such, that tissue specificity plays a role here as an explanation of why we see a pore-forming effect at 10 μM U_11_ and higher, in contrast to Barassé et al. (2023), who attribute neurotoxic features to the pharmacology of U_11_ despite the absence of any demonstrated molecular target, such as voltage-gated potassium channels. It is precisely because Barassé et al. have hypothesized the presence of a functional dyad in U_11_, putatively responsible for blocking a potassium conductance and, as such, explaining a neurotoxic mechanism of action for the observed paralysis, that we have thoroughly checked this possibility by testing a wide array of voltage-gated potassium channels. As illustrated in Figure 2, we could not find any neurotoxic effect caused by potassium channel blockade, nor did we find any effect on voltage-gated sodium channels. Since these two types of voltage-gated ion channels are crucially involved in muscle activity, we doubt whether U_11_ exerts its neurotoxic effect via modulation of these channels. Instead, our results favor interpreting the observed paralysis due to pore formation. To substantiate this further, we have compared the well-known pore-forming capacity of bee venom (*Apis mellifera*) in our *Xenopus laevis* bioassay. The effect evoked by the bee venom is very similar, if not identical, to the effect caused by U_11_. This result makes the composition of the membrane of the oocyte implausible as an explanation for the difference in interpretation of U_11_’s effect between Barassé et al. (2023) (i.e., U_11_ being neurotoxic) and our work presented here (i.e., U_11_ as a pore-forming peptide). An additional advantage of our electrophysiological comparison of U_11_ with *Apis mellifera* venom is the quantitative analysis of the shift in reversal potential made possible: as illustrated in Figure 4, the depolarizing shift of the reversal potential induced by U_11_ is not significantly different from the one induced by *Apis mellifera*. Based on the amphipathic property of melittin, assumed to be the most important pore-forming peptide in *Apis mellifera* venom, and its easy insertion into membranes by disrupting both natural and synthetic phospholipid bilayers yielding non-selective pores, it can be surmised that U_11_ acts in a similar manner with pores that are also non-selective (or very slightly selective) for the flux of ions they allow. It should be emphasized that the measurement of a reversal potential using a voltage clamp is an accurate and reliable methodology to assess the ion selectivity of an ion channel or pore. Furthermore, the observation that the chord conductance in the presence of both bee venom and U_11_ has increased dramatically indicates that the pore-forming molecules, in both cases, have generated a kind of ‘big gate’ rather than a ‘small door’. This helps to explain the fast kinetics of cytolysis observed.

As several ant venoms have paralytic effects on arthropods, it is generally believed that they also contain neurotoxins inducing paralysis. The two best-characterized neurotoxins from ants are (*i*) poneratoxin, a 25-amino-acid linear peptide from *Paraponera clavata* (Paraponerinae), which is a modulator of voltage-gated sodium channels [4]; and (*ii*) ectatomin Et-1 from the ant *Ectatomma tuberculatum*, a voltage-gated calcium channel blocker but also a pore-forming peptide [11]. In the case of *Tetramorium bicarinatum*, this most likely is also the case, based on the number of venom peptides found by Barassé et al. (2023) (U_2_–U_15_) with hitherto unknown function(s) but with clear paralysis-inducing effects when screened in a blowfly (*L. caesar*) paralytic assay. With this in mind, we do not claim that venom from the *Tetramorium bicarinatum* does not indeed contain one or more neuroactive peptides. Undoubtedly, future experiments will unravel the pharmacological effects contained in ant venom peptides, such as U_2_–U_15_ found in *Tetramorium bicarinatum* [33].

## Data Availability

Data, material, and software information are provided in the article.
